# International publication trends and collaboration performance of China in healthcare science and services research

**DOI:** 10.1186/s13584-016-0061-z

**Published:** 2016-01-29

**Authors:** Kai Chen, Qiang Yao, Ju Sun, Zhi-fei He, Lan Yao, Zhi-yong Liu

**Affiliations:** School of Medicine and Health Management, Tongji Medical College, Huazhong University of Science and Technology, Wuhan, Hubei 430030 China; School of Political Science and Public Administration, Wuhan University, Wuhan, Hubei 430072 People’s Republic of China

**Keywords:** Healthcare science and services, China, Publication trends, Collaboration patterns, Research topics, Web of Science

## Abstract

**Background:**

In recent years, China’s healthcare reforms and related studies have drawn particular global attention. The main objective of this study is to evaluate quantitatively the publication trends and collaboration performance of China in healthcare science and services (HSS) research.

**Methods:**

Scientometric methods and visualization technology were used to survey the growth and development trends of HSS research based on the Web of Science publications during the past 15 years.

**Results:**

China’s international publications on HSS research increased rapidly compared to those of the global HSS and Chinese scientific studies. Growth trends indicate that collaboration among countries, institutions and authors has also increased. China’s leading partners were all developed countries, such as the US, the UK, Australia and Canada, which have contributed to the majority of the joint publications. The academic impact of publications involving partners from European and American countries was relatively higher than those involving partners from Asian countries. Prominent institutions were universities that could be primarily classified into two groups, namely, Mainland China on the one hand and Hong Kong universities and foreign universities on the other. The most prominent actors were elite institutions, such as Peking University, Fudan University, Chinese University of Hong Kong, University of Hong Kong. The papers published by the Chinese Ministry of Health had relatively high academic impact, whereas those published by Mainland China universities alone had a lower academic impact compared to foreign cooperation papers. Issues related to the Chinese healthcare reform, priority diseases (e.g., breast cancer, HIV/AIDS, tuberculosis, etc.), health systems performance, quality of life and measurement tools, aging problems and research methods have been the most popular HSS topics in China in recent years.

**Conclusions:**

Despite the extensive achievement of the Chinese HSS reforms and research, gaps and challenges remain to be addressed, including those related to health insurance and the effects of the evaluation of essential medicine systems, human resources training and allocation in the health sector, government hospitals reforms and health services systems remodeling. These findings could help scholars and decision-makers understand the current status and likely future trends of the Chinese HSS research, and help them select the most appropriate collaboration partners and policies.

## Background

China has enjoyed rapid economic development since the reform and open policy implementation in 1978. Accordingly, China’s economic reforms have achieved significant success. However, the Chinese healthcare system began to regress in the 1980s, and many problems have been encountered thereafter. For example, from 1978 to 2012, China’s GDP increased at a 17.7 % compounded annual growth rate, whereas its total healthcare expenditure grew at a somewhat lower 15.7 % compounded annual growth rate [1]. With the economic reform initiated in the early 1980s in China, the healthcare system had to adapt to a new economic approach, namely, shifting from a communal system to market-driven competition.

Unfortunately, this disorientation caused several problems in Chinese society. The SARS outbreak in 2003 shocked the country’s leaders, thereby exposing the inadequacies in the public health protection system and demonstrating government negligence that had left the healthcare system unprepared to deal with its core responsibilities [2, 3]. Thereafter, the government struggled to maintain a balance between meeting the people’s immediate healthcare needs and developing the healthcare systems with a series of healthcare policies and reforms [4–6]. For example, after three years of planning, China unveiled its ambitious healthcare reform plan in April 2009 by committing an additional CN¥850 billion (approximately US$125 billion) over a three-year period with the provisional goal of affordable and equitable basic healthcare for all by 2020 [3, 7]. Influenced by health policies and economic investments, healthcare science and services (HSS) research in China began to escalate and develop rapidly.

With the increase in HSS research in China, research collaboration also proliferated in this field. Scientists benefited from intellectual exchanges with foreign colleagues and reduced costs by sharing resources and technologies with other countries [8]. Apart from improving research capability, international collaboration may enhance productivity and visibility, although visibility improvement varies among countries and fields [9]. In recent decades, the robust relationship between collaboration and scientific research productivity and academic impact has been studied and documented by Lotka [10] and others. In general, collaboration exerts positive effects on teams’ outputs and abilities, and cooperative scientific research results have relatively high academic impact, particularly those related to international collaboration. For example, the citations of a paper are partially related to the number of authors, institutions and countries participating in the paper. However, the effects depend on the collaboration type and the partners involved [11–13]. For example, Narin showed that multiple-institution papers are more highly cited than single-institution papers, and papers with a foreign collaborator are more highly cited than domestic papers [11, 14]. Glänzel demonstrated that the influence of international collaboration on national citation performance also varies considerably between countries. In some cases, no quality advantage exists for one or both partners, such as in certain collaborations among developing or Eastern European countries [15]. Meanwhile, the collaboration patterns and influences on research productivity or academic impact also vary by discipline [13, 15]. Therefore, understanding the collaboration characteristics of specific fields could inform the policies on partners’ selection and research performance improvements, and could even contribute to economic development [16, 17].

This study was built on our previous work which analyzed the global progress and current research trends on health care sciences and services research [18]. Although the rapid growth and extensive collaboration in HSS research is observed in theory and practice in China, the publication trends, collaboration patterns and their effects on HSS research in this country remain unclear. Limited attention has also been focused on which groups of actors are at the center and at the periphery of the collaboration network. The previous study was a comprehensive scientomentric research from a global perspectives, while this study focuses on China's collaboration relationships in HSS research. Both collaboration relationship and publication patterns were studied on multiple levels and from several perspectives. With the availability of critical data, such as those from the Web of Science (WoS) of Thomson Reuters, scientometricians have attempted to explore the characteristics of international collaboration from various perspectives [9]. Given the importance of Chinese collaboration in the international context, the current study evaluated the publication trends, collaboration patterns and current research trends of Chinese HSS.

Thus, the objectives of this study are as follows: (1) to study the Chinese HSS publication trends from absolute and relative perspectives, (2) to explore the collaboration patterns and identify the core partners and institutions, and (3) to present the research foci of HSS in China. The results of this study could provide evidence on the current status and recent trends of publication and collaboration in China, as well as indications of this topic’s popularity and citation performance.

## Methods

### Data sources

This study was based on the analysis of article-level data from the online version of the WoS database, which is owned by Thomson Reuters. WoS is a highly significant and frequently employed source database in reviewing scientific achievements and trends [19, 20]. Therefore, HSS-related articles from WoS were suitable for the present study. Data in this study were acquired on February 2, 2015 using the following search strategy: *SU = Health Care Sciences & Services AND CU = China AND PY* = 2000–2014. A total of 2416 related papers were extracted from the databases. Thereafter, the bibliographies were downloaded and imported into a bibliographic software program. The *Health Care Sciences & Services* subject contains healthcare science, healthcare services and health policy-relevant research within the WoS database.

### Analysis Methods

Scientometric methods have been extensively used recently to analyze scientific productions, collaborations and research topics [19, 21–29]. Scientometric and related indicators are also suitable for scientific literature analysis from both the macro- and micro-perspectives. In the present study, the performance of the publication and collaboration of HSS research in China is analyzed from both the quantity and academic impact perspectives. Quantity was determined by the number of publications and the growth trends were measured through two related parameters, namely, relative growth rate (RGR) and doubling time (Dt) [30]. The academic impact of the papers was measured using the total local citation score (TLCS), total global citation score (TGCS), and average global citation score (AGCS) [31]. Co-authorship and social network analysis were also used to study the collaboration at the country and institution levels [18]. Furthermore, co-words and cluster analysis were used in combination to identify the popular topics. Visualization technology, particularly knowledge mapping technology, was also used to show the results of the collaboration between countries or institutions and popular research topics. Thomson Data Analyzer (TDA) [32] and HistCite [33] were used as statistical analysis tools. The drawing tools used in this study include Ucinet [34] and VOSviewer [35].

#### Growth speed indicators

RGR is originally sourced from the study of financial investment and is effectively applied in botany to analyze the growth of individual plants [36]. In the current study, RGR was used to measure the growth rate of the number of articles with time. Meanwhile, Dt is directly related to RGR and is the time required for the number of articles to double [26, 29, 37]. RGR and Dt are defined as follows:RGR = (lnN2 ‐ lnN2)/(*t*2 − *t*1) (Formula 1) *Dt* = (*t*2 − *t*1) * *ln*2/(*lnN*2 − l*n N*1) (Formula 2),where N2 and N1 are the cumulative publications in two years, that is, t2 and t1, respectively. In the present analysis, t2 − t1 is considered 1 year. Thereafter, RGR and Dt can be expressed as *RGR* = *ln* (*N*2/*N*1) and *Dt* = *In*2/*RGR*. A constant value for RGR in each subsequent year is an indication that the growth rate is exponential. Dt is a characteristic time for this exponential growth.

#### Academic impact indicators

The academic impact indicators used in this study include TLCS, TGCS and AGCS based on citation frequency. TLCS is the number of times that papers in a set included in a collection has been cited by other papers *within the collection*. TGCS is the number of times that papers in a set included in a collection has been cited *in the WoS database*. AGCS is the mean value of TGCS and indicates the average citation number of articles in the HSS areas. TLCS and TGCS have been the key indicators in evaluating the relevance of research papers [18].

#### Collaboration performance indicators

The term “co-authorship” is often used to denote multiple authors, institutions or countries appearing simultaneously in one paper; in this article, they are called co-authors, co-institutions and co-nations, respectively. Meanwhile, social network analysis (SNA) and the related centrality indicators were also used to analyze the collaboration performance of institutions and countries. As a “structural analysis” method, SNA has been successfully applied in various fields, such as sociology, information and library sciences, geography and other areas [38]. SNA has also been extensively used to investigate scientific collaboration networks and the relationship between individuals at the author, institution and country levels [17, 39, 40]. The current study analyzes trends in the extent of collaboration at all levels and the collaboration networks at the country and institution levels, because country and institution collaboration can reveal the collaboration at the macro- and meso-levels, respectively. The combination of the two levels can facilitate further understanding of trends, networks and core groups of international collaboration [41]. In the present study, network nodes represent institutions or countries, whereas ties represent the cooperation of institutions or countries. Degree centrality is defined as the number of ties of a node, representing the simplest notion of centrality because such a value simply refers to the number of neighbors of a node in the network. Degree centrality is a crucial indicator in analyzing the network, thereby reflecting the importance and influence of an institution or a country in the network [18].

## Results

### Growth trends from multiple perspectives

Table 1 shows the year-wise publications of the world and China in the HSS area, as well as those of China in all fields, including the year number, cumulative number, RGR and Dt from 2000 to 2014.

**Table 1:** Chinese and worldwide publication trends in healthcare science and services, 2000-2014

Year	No. of China	Cumulative China	No. of World	Cumulative World	No. of China all	Cumulative China all	RGR China	RGR World	RGR China all	Dt China	Dt World	Dt China all
2000	37	37	7730	7730	31953	31953	—	—	—	—	—	—
2001	43	80	6814	14544	37407	69360	0.77	0.78	0.16	0.90	0.89	4.40
2002	48	128	8235	22779	42316	111676	0.47	0.48	0.12	1.47	1.46	5.62
2003	55	183	8903	31682	51843	163519	0.36	0.38	0.20	1.94	1.82	3.41
2004	63	246	9162	40844	65036	228555	0.30	0.33	0.23	2.34	2.07	3.06
2005	54	300	9949	50793	76268	304823	0.20	0.29	0.16	3.49	2.41	4.35
2006	69	369	10920	61713	92630	397453	0.21	0.27	0.19	3.35	2.61	3.57
2007	87	456	11570	73283	100981	498434	0.21	0.23	0.09	3.27	3.06	8.03
2008	124	580	12775	86058	117710	616144	0.24	0.21	0.15	2.88	3.27	4.52
2009	159	739	14272	100330	134307	750451	0.24	0.20	0.13	2.86	3.52	5.25
2010	223	962	14618	114948	149457	899908	0.26	0.18	0.11	2.63	3.82	6.49
2011	228	1190	15921	130869	173125	1073033	0.21	0.18	0.15	3.26	3.94	4.72
2012	351	1541	18337	149206	199132	1272165	0.26	0.17	0.14	2.68	4.07	4.95
2013	348	1889	19317	168523	236653	1508818	0.20	0.17	0.17	3.40	4.06	4.02
2014	527	2416	19307	187830	264943	1773761	0.25	0.16	0.11	2.82	4.28	6.14

No. of China: number of articles published by China on HSS; No. of World: number of articles published by the world on HSS; No. of China all: number of articles published by China on all fields

#### Absolute perspective

Table 1 presents the number of papers on HSS in China and in the world from 2000 to 2014. During the past two decades, the number of published papers related to HSS increased from 37 in 2000 to above 500 in 2014. Table 1 also shows that only a few papers were produced before 2005, which was the formative stage of the Chinese HSS research. From 2005 to 2010, which was the development stage of Chinese HSS research, the annual output of papers did not exceed 200. Thereafter, the number of papers increased rapidly (i.e., after 2010), when the Chinese HSS research entered the rapid development stage. Nevertheless, the inflection point of maturity, where growth begins to taper off, has not yet been reached.

#### Relative perspective

**Fig. 1:**
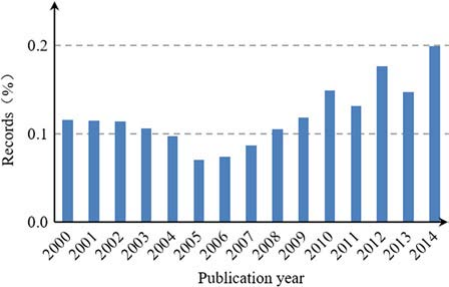
Chinese HSS papers as a percentage of Chinese papers in all fields

**Fig. 2:**
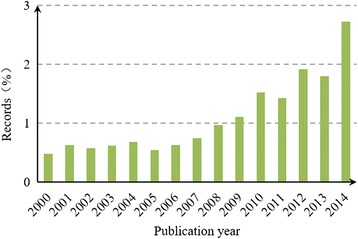
Chinese HSS papers as a percentage of the world HSS papers

Figures 1 and 2 show the Chinese HSS papers as a percentage of Chinese papers in all fields and the world HSS papers. The percentage of Chinese HSS papers reveals the publication trends from a relative perspective, which can precisely and relatively reflect the development trends of Chinese HSS research. The percentage of Chinese HSS papers exhibited up and down trends, thereby implying a decrease and a rapid increase, respectively, before and after 2005. The increasing stage could be divided into two stages, namely, constant growth (2005–2010) and fluctuant growth (2010–2014) stages. These results are considered based on the publication trends, thereby suggesting that Chinese researchers focused considerable attention and effort to HSS studies compared to other fields in China and to HSS research in the world.

#### Dynamic perspective

The growth perspective indicates that the average values of worldwide RGR and Dt were 0.29 and 2.95, respectively. The average values of RGR and Dt for the Chinese HSS research were 0.30 and 2.66, respectively (Table 1). Meanwhile, the average values of RGR and Dt for all fields in China were 0.15 and 4.89. In the HSS field, the global growth rate showed a significant decrease during the last 15 years. Meanwhile, China revealed up and down trends that indicate a decrease during the first 5 years (from 2001 to 2005), which was a constant trend after 2005 and a trend exceeding that of the world in 2008. The growth rates of HSS research in both China and the world were higher than those of all fields in China. In addition, the growth rate of HSS research in China showed an impressive increase of 21 % during the last 15 years. This figure was significantly higher than that of HSS research in the world (7 %) and of all fields in China (16 %).

### Collaboration and performance

#### Collaboration trends

**Fig. 3:**
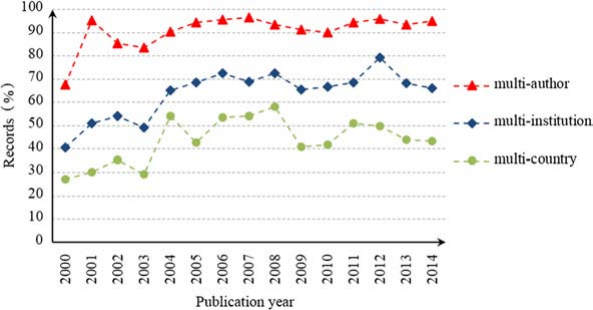
Percentage of multi-entity in Chinese HSS papers from 2000 to 2014

Chinese collaboration with other countries in HSS research exhibits up and down trends; however, the general tendency of the trends is to increase, particularly after 2003. Figure 3 shows the percentage of collaborative papers, including those involving co-authors, co-institutions or co-countries. The percentage of co-authored papers increased from 68 % in 2000 to 95 % in 2014. The percentages of co-institutional papers and co-national papers showed increasing trends from 41 to 67 % and from 27 to 43 %, respectively. These results suggest that the collaboration between institutions and countries has increased significantly since 2003.

**Fig. 4:**
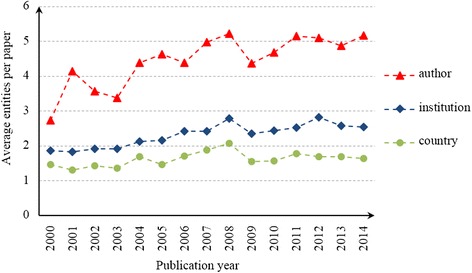
Average number of different entities per paper in Chinese HSS papers from 2000 to 2014

Figure 4 shows the trends of the average number of authors, institutions or countries per paper. The average number of authors per paper increased from 2.73 in 2000 to 5.16 in 2014, whereas the average number of countries per paper only increased from 1.46 in 2000 to 1.64 in 2014. More than half of the papers are the result of the cooperation within China; hence, these papers have an average of approximately two institutions and four to five authors.

#### Partner Countries

In terms of quantity, China collaborated with 84 countries in HSS research over the last 15 years. Among China’s partners, the US played a major role, accounting for approximately one-fourth of the Chinese joint publications in HSS. The UK, the second largest partner of China, was left significantly behind. Australia and Canada were also important partners of China. Overall, China’s top 10 partners contributed to more than half of the joint publications. In terms of academic impact, collaborations with the US has the highest TGCS because of the numerous papers published, followed by collaborations with the UK, Australia and Canada. The AGCS of countries are listed in descending order and includes those of the Netherlands, Switzerland, the UK and Japan, among others. Papers from China which were written in collaboration with these countries often have considerably high academic impact and influence (Table 2).

**Table 2:** Top 10 productive partners of China in healthcare science and services, 2000-2014

Rank	Partner	Joint Publications	Percent	Recs 1st	Percent 1st	TLCS	TGCS	AGCS
1	US	566	23.43	265	46.82	316	3477	6.14
2	UK	172	7.12	58	33.72	124	1523	8.85
3	Australia	149	6.17	51	34.23	63	898	6.03
4	Canada	84	3.48	31	36.90	40	653	7.77
5	Singapore	53	2.19	21	39.62	9	163	3.08
6	Taiwan	46	1.90	23	50.00	2	143	3.11
7	Japan	45	1.86	15	33.33	20	378	8.40
8	Switzerland	45	1.86	12	26.67	34	459	10.20
9	The Netherlands	38	1.57	10	26.32	5	418	11.00
10	Germany	35	1.45	10	28.57	3	159	4.54

Joint Publications: number of articles collaborated with China; Percent: percentage of articles; Recs 1st: number of articles collaborated with China as first country; Percent 1st: percentage of articles collaborated with China as first country; TLCS: Total Local Citation Score, which is the number of times cited by other papers in the local collection; TGCS: Total Global Citation Score, which is the citation frequency based on the full WoS count at the time the data were downloaded; AGCS is the average citation frequency of an article

**Fig. 5:**
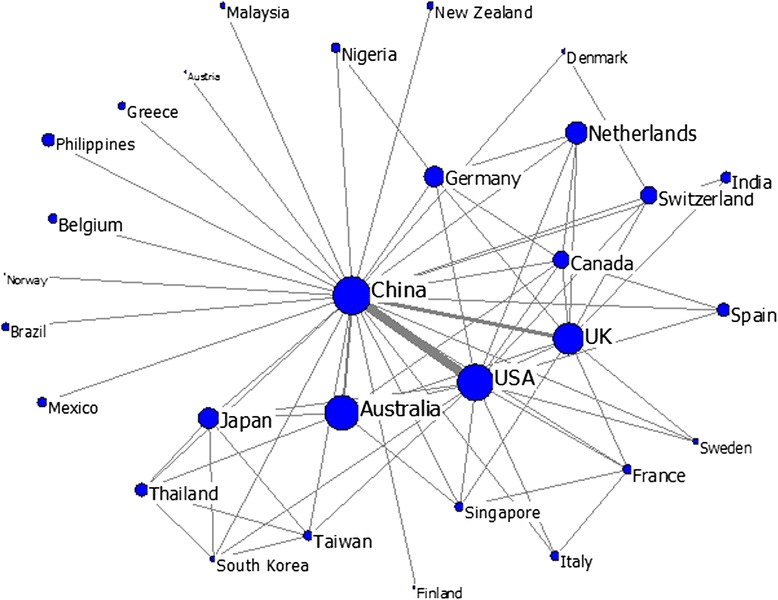
National collaboration map of China in HSS research (The size of the nodes represents the degree centrality of the countries, and the thickness of the lines represents the co-occurrence frequency between countries)

The collaboration relationships among a core group of countries (top 30) were visualized using Ucinet, which allowed for a number of analytical procedures to be undertaken to determine the types of shared relationships among the countries or regions [42]. Fig. 5 presents the national or regional cooperative relationship with China in HSS research. In this figure, the size of the nodes stands for the centrality degree of a specific country (i.e. the extent to which it is involved in HSS publications related to China), whereas the thickness of the links stands for the collaboration strength between countries. China is located at the core position in the network and cooperates frequently with the US, the UK, Australia, Canada, Germany, Netherlands, Switzerland and Japan, among others. Meanwhile, Taiwan, Singapore and other countries are located at the periphery. This situation means that China’s cooperation with this second group of countries in studying HSS research is not as frequent as that with the first group. Other countries or regions located in the outermost loci of the cooperation network cooperate less with China in this area.

#### Prominent institutions

One thousand seven hundred eighty-nine institutions contributed to the set of publications considered in this study. Table 3 shows the top 20 institutions, each of which has published over 30 papers, and which together account for over two-thirds of all publications. The Chinese University of Hong Kong (CUHK) performed extremely well and significantly exceeded other institutions with a total of 333 papers published. This finding suggests that CUHK is the most prominent institution in terms of HSS research among Chinese institutions. CUHK is followed by the University of Hong Kong, Peking University, Fudan University and other universities. Among non-academic institutions, China’s Ministry of Health had the highest citations per paper and substantially exceeded other institutions. This ministry is followed by Harvard University, Wayne State University and Shandong University. Meanwhile, CUHK, University of Hong Kong, Chinese Centre for Disease Control and Prevention (CDC), and Prince Wales Hospital also had relatively high AGCSs. The AGCSs of articles published solely by researchers from Mainland China’s institutions were relatively low, thereby indicating that the articles produced in cooperation with foreign institutions, particularly the prominent ones, have relatively high academic impact.

**Table 3:** Top 20 productive institutions with China in healthcare science and services, 2000-2014

No.	Institution	Recs	Percent	Recs 1st	Percent 1st	TLCS	TGCS	AGCS
1	Chinese Univ Hong Kong	333	13.78	260	78.08	144	2537	7.62
2	Univ Hong Kong	255	10.55	195	76.47	154	1938	7.60
3	Peking Univ	222	9.19	119	53.60	91	1005	4.53
4	Fudan Univ	156	6.46	103	66.03	63	731	4.69
5	Sichuan Univ	81	3.35	54	66.67	14	235	2.90
6	Hong Kong Polytech Univ	79	3.27	55	69.62	24	392	4.96
7	Zhejiang Univ	78	3.23	57	73.08	16	139	1.78
8	Huazhong Univ Sci & Technol	57	2.36	43	75.44	30	200	3.50
9	Sun Yat Sen Univ	56	2.32	28	50.00	23	247	4.41
10	Shandong Univ	47	1.95	26	55.32	78	385	8.19
11	Chinese Ctr Dis Control & Prevent	45	1.86	29	64.44	21	334	7.42
12	Shanghai Jiao Tong Univ	44	1.82	26	59.09	23	203	4.61
13	Wayne State Univ	39	1.61	15	38.46	48	345	8.85
14	Minist Hlth	38	1.57	8	21.05	90	569	14.97
15	Prince Wales Hosp	37	1.53	9	24.32	23	259	7.00
16	Xi An Jiao Tong Univ	36	1.49	23	63.89	7	76	2.11
17	Second Mil Med Univ	35	1.45	29	82.86	11	134	3.83
18	Harvard Univ	34	1.41	7	20.59	54	339	9.97
19	Nanjing Med Univ	34	1.41	18	52.94	9	134	3.94
20	Natl Univ Singapore	34	1.41	14	41.18	9	109	3.21

Recs: number of articles; Percent: percentage of articles; Recs 1st: number of articles as first institution; Percent 1st: percentage of articles as first institution; TLCS: Total Local Citation Score, which is the number of times cited by other papers in the local collection; TGCS: Total Global Citation Score, which is the citation frequency based on the full WoS count at the time the data were downloaded; AGCS is the average citation frequency of an article

**Fig. 6:**
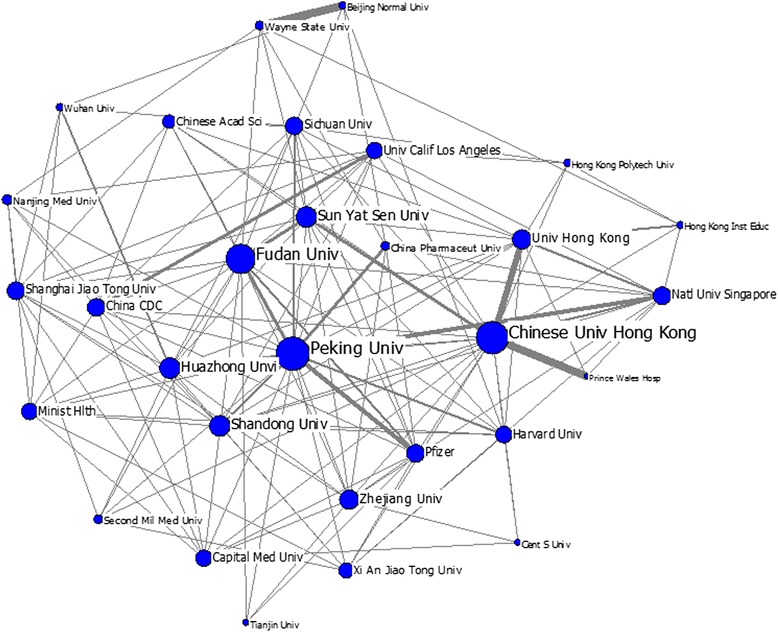
Institutional collaboration map of China in HSS research (The size of the nodes represents the degree centrality of the institutions, and the thickness of the lines represents the co-occurrence frequency between institutions)

Figure 6 represents the mapping of the top 30 institutions involved in the cooperation of the network in HSS research with China. Chinese Peking University, Fudan University and University of Hong Kong are evidently in the core of the network, thereby indicating that these institutions cooperate with considerable frequency with other academic institutions. This finding also means that these institutions play a significant role in the process of HSS knowledge transfer on a global scale. Meanwhile, the entire network can be divided into two institutional groups of cooperation. One group mainly comprises institutions in Mainland China, such as Peking University, Fudan University, Huazhong University of Science and Technology, Shandong University, Sun Yat Sen University and the Chinese CDC, among others. The other group primarily comprises various Hong Kong institutions, such as CUHK, University of Hong Kong, Hong Kong Polytechnic University, Hong Kong Baptist University, City University of Hong Kong, Hong Kong Institute of Education; and a few foreign institutions, such as Harvard University and National University of Singapore.

### Research topics

**Fig. 7:**
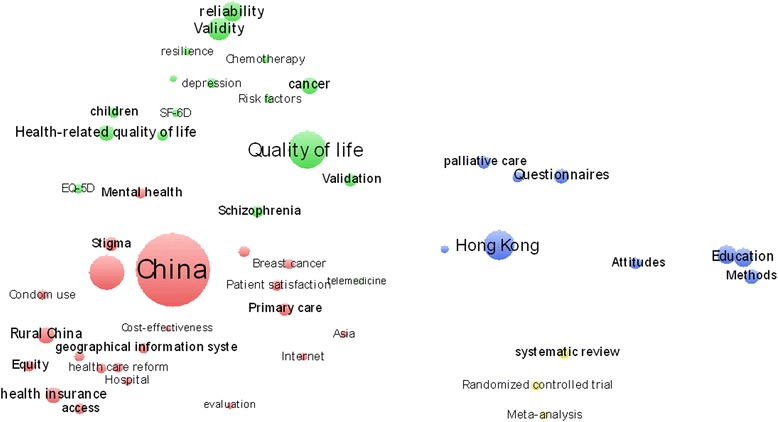
Co-occurrence clustering map of author keywords (The size of the nodes indicates the frequency of the keywords, and the distance between two nodes is inversely proportional to the number of co-occurrence between keywords, that is, shorter distances suggest greater co-occurrence between keywords)

The authors’ keywords could offer information on how the authors conceptualize their own research, and such keywords have been proven vital in monitoring the development of science [43–45]. Therefore, the papers’ topics can be observed from the authors’ keywords through cluster analysis. The 55 authors’ keywords are divided into four groups that represent popular research topics on HSS research in China (Fig. 7).

Group 1 (red) – Chinese health topics, including healthcare reform-related topics (e.g., health insurance, primary care, rural health, hospital problems), priority diseases or public health problems, health systems performance and Chinese cultural questions for health, are included in this group. In the past decades, healthcare reforms were the major issues in China. Various policies adopted by health policymakers indicate that China’s healthcare system reform passed through three periods [46] and numerous milestone events also happened during these periods [3–5, 7] (For details, see Appendix 1). Meanwhile, setting the priority areas and evaluating the effects of health reform are two vital topics in the health reform process. Accordingly, it is not surprising that articles on topics related to the Chinese health care reform figure prominently among Chinese HSS publications.

The analysis also indicates that Chinese HSS publications also focus on specific diseases and health issues, such as breast cancer, mental health, HIV/AIDS, tuberculosis, schistosomiasis and condom use, among others. Furthermore, healthcare system performance assessment is an important tool used to evaluate the effects and performance of healthcare reforms. To monitor and evaluate the reforms effects or health system status, the Chinese government and its universities have done a lot of research on health systems performance assessments. As showing in Fig. 7, Chinese healthcare systems may be evaluated from the aspects of health equity, health access, health cost effectiveness, patient satisfaction and other aspects.

Group 2 (green) – Quality of life (QOL)-related topics, such as the measurement of QOL or health-related quality of life, QOL measurement tools and reliability and validity of the QOL measurement tools, are included in this group. The QOL outcomes are significant topics in HSS research. The World Health Organization (WHO) defines QOL as an “individual’s perception of their position in life in the context of the culture and value systems in which they live and in relation to their goals, expectations, standards and concerns” [47]. Economic development has led the Chinese to focus considerable attention on QOL. Therefore, QOL is a crucial research topic in China, particularly in recent years. Chinese researchers mainly focus on developing or improving the measurement tools based on the world’s popular tools and Chinese situations, such as SF-36, SF-12, SF-6D, WHOQOL and EQ-5D [48]. Researchers often use reliability and validity indicators to evaluate the effects of the developed or modified tools. Thereafter, they can measure and understand the Chinese’s QOL and even compare it with that of people from other countries. QOL research mainly focuses on children and the aging population [49, 50]. Significant attention has also been focused on QOL of patients with cancer, schizophrenia and depression [51, 52].

Group 3 (blue) – Topics related to the elderly, including palliative or end-of-life care and medical education and methods, are included in this group. Hong Kong researchers devote significant attention to the problems of old people, such as old people’s QOL, disability trends [53], lifestyle risk factors [54], willingness to pay for specific primary care and preventive services [55], and others. Mainland China and Hong Kong closely cooperate in the area of palliative or end-of-life care. Palliative care provides relief from pain and distressing symptoms, as well as offers psychological and spiritual support to enhance patients’ QOL. Meanwhile, the needs, current knowledge and attitudes of old people who come from staffed homes in Hong Kong are marked differently [56]. Studies in Hong Kong determined that cancer patients have a relatively high level of palliative care, which has played a role in improving end-of-life cancer care in this city [57, 58]. Developing palliative care for end-stage diseases has also attracted the attention of Hong Kong researchers [59]. Both Mainland China and Hong Kong attach importance to medical education, as observed in the new medical curriculum evaluation [60], evidence-based medicine practice, barriers identification and teaching/learning assessment [61, 62].

Group 4 (yellow) – Research methods, including systematic review, meta-analysis and randomized controlled trial, are included in this group. Systematic review and meta-analysis have been extensively used by Chinese researchers in HSS. Moreover, an increasing number of Chinese researchers tend to use these methods to write articles because of their advantage in publishing science citation index/social sciences citation index (SCI/SSCI) articles. Several researchers also devote attention to the bias in meta-analysis and attempt to test and modify this method [63, 64].

## Discussion

### Good performance and bright future from multiple growth perspectives

China has performed well in the HSS field, thereby indicating a continuously rising trend that has increased exponentially in the last 15 years. The growth rate of HSS research in China is significantly faster than that of HSS research in the world and of other fields in China. Recent trends in Chinese HSS research generally suggest a bright future in terms of quantity compared to the HSS research in the world and with other fields in China.

Many factors may have contributed to the rapid growth of international Chinese publications in the HSS fields, such as health policies and reforms, health projects and funds, international collaboration, research evaluation policies or mechanisms, graduate student enrolment expansion and other factors. First, the Chinese healthcare reforms in the HSS field commenced in the 1980s and China has undergone three reform periods [46]. Meanwhile, numerous milestone events occurred during these periods. In 2006, health officials and decision-makers realized that the previous reforms were unsuccessful; thus, a new round of reforms began [4, 5]. In April 2009, after three years of planning, China unveiled its ambitious healthcare reform plan by committing to invest an additional CN¥850 billion (approximately US$125 billion) over a three-year period with the provisional goal of providing affordable and equitable basic health care for all by 2020 [3, 7]. Therefore, these remarkable events have been highly considered by scholars and healthcare institutions, as well as contributed to the growth of Chinese HSS research.

Second, the consistent increase in investments in various healthcare fields enabled China to launch additional health projects and provide funds to support Chinese researchers in conducting high academic impact research. For example, the National Natural Science Fund and the Ministry of Health of China established health management and policy category funds to support HSS research; the financing amount for this program increases annually.

Third, the collaboration between China and other countries or international institutions has become increasingly frequent. The WHO, World Bank (WB) and China Medical Board (CMB) provide training and funds to Chinese researchers. For example, the WB/DFID China Rural Health Project (2008 to 2013) is a typical (and very large) program, which is funded by the Chinese government, a WB loan and a grant from the UK Department of International Development (DFID) [65]. This project aims to provide experience and a model for promoting rural health reform in areas in China with different development levels. During this process, WB and the UK government supplied financial and technical/academic support for China. CMB also supported Chinese researchers in universities to study health policy and system science-related fields [66]. These international activities and collaborations may assist Chinese researchers to enhance their research skills and publish additional international papers.

Chinese research evaluation policies also encourage students to publish international papers. This case is evident in the increasing number of universities requiring additional staff members and doctoral and master’s students to publish SCI/SSCI papers as one of their requirements for graduation [67]. Therefore, the number of international papers increased with the expansion of graduate studies enrolment.

### Collaboration between Chinese and foreign researchers is becoming increasingly frequent

The collaboration trends between countries, institutions and authors develop along with the publication growth trends. Moreover, the cooperation of authors is evidently higher than that of institutions and countries. Therefore, Chinese research capacity and international influence in HSS research may continue to strengthen. However, the collaboration of authors is mostly conducted within institutions or countries. The collaboration of China with over two countries in one paper is still infrequent. Given that productivity and are positively related to extent and patterns of collaboration s, Chinese researchers should improve the extent of collaboration with foreign countries and institutions, particularly with developed countries and well-known institutions.

### Collaboration and performance with core partners and institutions

China’s leading partners in HSS research are developed countries, with the quantity of papers resulting from cooperation with the US and the UK leading the way. The academic impact of papers is also generally high for European countries, such as Switzerland, the Netherlands and others. Therefore, international collaboration with these partners could improve the academic impact of Chinese HSS research output. Hence, international collaboration is still necessary for China to enhance its HSS research capacity. Meanwhile, the quantity of papers resulting in cooperation with Asian countries is relatively low, and the academic impact of these papers also needs improvement. The aforementioned situation can also be observed in the analysis of collaboration networks; hence, developed countries have conducted considerable research in this field with China.

This finding is consistent with parallel findings regarding regular scientific research, which is positively correlated with the level of economic development [68]. As a developing country with a rapidly expanding economy in the last 15 years, China’s strategy to solve its domestic health problems involves utilizing its high output in the health reform domain and cooperating with several developed countries for its health reform needs. Therefore, China could strengthen its collaboration relationship with the aforementioned countries to improve the academic impact of research. In this process, Chinese researchers could also learn advanced methods and valuable experience.

The concentration of HSS research in China is primarily based in universities. Among the top 20 institutions in this field, 17 are universities and the remaining 3 are R&D organizations. A few elite Chinese institutions, including CUHK, University of Hong Kong, Peking University and Fudan University, contributed approximately half of the Chinese studies and have collaborated with many international institutions. Additionally, the collaboration between the institutions of Mainland China and Hong Kong was frequent and close; however, the international collaboration of the former’s institutions was weaker than that of latter’s. Moreover, the papers published by China’s Ministry of Health have a relatively high academic impact because it is a trusted source of information, and most of the ministry’s papers were developed in collaboration with the US and the UK. However, papers from Mainland China are considered low academic impact papers. Mainland China merely has seven institutions, whereas numerous Hong Kong institutions play an important role in Chinese HSS research. Therefore, Mainland China’s institutions should consider strengthening their collaborations with renowned institutions in the HSS field.

### Popular research topics are associated with China’s healthcare reforms

The analysis of keywords shows that popular Chinese health reform-centered topics were studied during the last decades. In spite of its extensive range, HSS research in China relatively focuses on healthcare reform, priority diseases (e.g., breast cancer, HIV/AIDS, tuberculosis, schistosomiasis, etc.), health systems performance, QOL and measurement tools, palliative or end-of-life care, old people problems, research methods and other related topics, particularly those associated to China. These topics drew significant attention from researchers and policymakers.

Healthcare system reform in China has been the most important topic during the last 30 years, particularly after the latest reform in 2009. China’s healthcare system reform has achieved major milestones, particularly in health insurance, under the government’s leadership. However, further analysis of the popular topics of the related articles highlighted gaps and challenges that need to be addressed to achieve China’s stated reform goals. First, despite the extensive achievement in the coverage of health insurance in China, particularly the New Cooperative and Medical Scheme (NCMS) and Urban Resident Basic Medical Insurance (URBMI), the effects of health insurance on the reduction of financial risks is still unclear [69–72]. Second, although the objective of the pharmaceutical sector to control drugs is correct, the methods and the effectiveness of such methods are unclear [73, 74] and relatively little has been published in this area. Third, human resources shortage exists in China; hence, this problem is a major obstacle in strengthening the public health and primary health care of the country [75]. In addition, retaining qualified health professionals in the rural areas, particularly in the poor and underdeveloped regions, has been difficult because of socioeconomic reasons and the lack of a proper healthcare infrastructure [3]. Even with the availability of staff members, the lack of incentives (e.g., higher wages, work benefits, paid vacations, etc.) for primary healthcare workers to deliver public health services may be the main hindrance in retaining healthcare human resources in the rural areas. Fourth, reforming public hospitals is one of the key issues in controlling the increase in healthcare expenditures, improvement in the academic impact of care, and reduction of waste and inefficient work performance [3]. The reform of public hospitals, particularly county and city government hospitals, needs significant attention and considerable effort to improve the academic impact of service delivery. Finally, health services systems (including provision and utilization) in China are disorganized, and the referral systems and the function orientation of different types of hospitals are still unclear. Relatively little has been published on these important topics to date, so these fields may be important frontiers for future Chinese HSS research.

## Conclusions

This study shows the publication trends, collaboration patterns and research foci of HSS research in China form multiple perspectives and levels. These results could assist both Chinese and foreign researchers, decision-makers and students understand the collaboration networks, select research themes and determine their partners in HSS research. Therefore, policymakers and researchers could foster a promising type of international collaboration network and improve the research performance for HSS research related to China.

## Appendix 1

### The history and milestone of Chinese healthcare reforms from 1980 to 2015

In the past decades, healthcare reforms were the major issues in China. Various policies adopted by health policymakers indicate that China’s healthcare system reform passed through three periods [46] classified as follows: (1) 1980s to 1993: market participation; (2) 1994–2005: market orientation (profound marketization); and (3) 2006 to present: government-driven market involvement. Numerous milestone events also happened during these periods. For example, China’s healthcare system reform began from the early 1980s to 1985. This period was regarded as the first stage of the reforms because the government officially approved the implementation of the healthcare sector reform. Healthcare financing was transferred from the central government to the local governments. In 1990, many discussions were conducted focusing on the dominant actor that should supervise the implementation of the healthcare system reform. This implementation was determined by the central government that set the main principles for “market participation” in 1992. In 1994, China began to reduce expenditures in the healthcare system. In 1997, the Chinese government encouraged the healthcare market to become market-oriented. In 2000, the privatization of several hospitals was allowed, thereby resulting in the emergence of many private hospitals since then. In 2006, health officials and decision-makers realized that the previous reform was unsuccessful; thus, they implemented a new round of reforms [4, 5]. In April 2009, China unveiled its new healthcare reform plan with the provisional goal of providing affordable and equitable basic health care for all by 2020 [3, 7]. This reform was anchored on five interdependent aspects: expanding healthcare coverage to over 90 % of the population, establishing a national essential medicine system to meet all the citizens’ primary needs for medicine, improving primary care delivery system to provide basic health care and to manage referrals to hospitals’ specialists, making public health services available and equitable to all, and steering public hospitals reforms [7, 76, 77]. Before announcing the new healthcare system reform, the Chinese government was faced with widespread public discontent that stemmed from the following factors: unaffordable access to health care, major financial risks associated with out-of-pocket health expenditures, and growing inequalities in accessing healthcare and health services across populations with different socioeconomic status, as well as between urban and rural areas [78–83]. Meanwhile, a few of the previously eradicated infectious diseases had re-emerged, whereas non-communicable diseases (NCDs) increased unabatedly [84–86]. In 2003, the SARS outbreak shocked China’s leaders, thereby exposing the inadequacies in the public health protection system and demonstrating government negligence that had left the health system unprepared to deal with its core responsibilities [2, 3]. Hence, examining the reform priorities against the problems and their causes in the pre-reform system led health authorities to determine that the current reforms are considerably headed in the right direction. The next phase of reforms, which was announced in detail in 2012 and 2013, intended to further improve the 2009 reforms [87, 88]. The 2015 health reform focuses on promoting government hospital reform (including county and city hospitals), thereby improving the universal health insurance system, strengthening the essential medications system, improving the pharmaceutical supply system, promoting an equitable primary public health services, establishing hierarchical medical systems and referral mechanisms, establishing proper health worker training and wage payment systems, promoting the development of the health services industry, and accelerating the development of population health information systems [89].
